# Unusual side effects with acamprosate

**DOI:** 10.4103/0019-5545.33266

**Published:** 2007

**Authors:** Ajeet Kumar Sidana, Divya Mangla

**Affiliations:** Department of Psychiatry, Government Medical College Hospital, Sector-32, Chandigarh - 160 047, India. E-mail: ajeetsidana@hotmail.com

Sir,

Acamprosate is a calcium acetylhomotaurine or calcium acetylaminopropane sulphonate and its molecular structure is related to that of GABA.[1] Acamprosate reduces the craving in detoxified alcohol dependence syndrome patients by reducing glutamate action at both pre- and postsynaptic levels and enhancing GABA function.[2] Major side effects reported are gastro-intestinal (25.1%), dermatological (9.1%), muscular (8.3%), tiredness (12.8), genito-urinary / sexual (37.5%), neurological / psychological (37.5%) and cardio / pulmonary (7.6%).[3] The drug has been available in the Indian market since more than three years but has been approved only recently by the US Food and Drug Administration (FDA) for the treatment of alcoholism.[4] Here, the authors have reported a case of unusual and troublesome side effects with acamprosate therapy in a detoxified alcohol dependence syndrome patient.

A 36 year-old, married, employed graduate, hailing from a middle socio-economic background and having a history of regular alcohol intake for the last four years, was diagnosed as a case of Alcohol Dependence syndrome as per the International Classification of Diseases (ICD-10).[5] He was detoxified with Lorazepam 6 mg/day for one week as an inpatient. The baseline renal function tests were within normal limits (Blood Urea = 20 mg/dl, Serum Creatinine = 0.8 mg/dl). He was put on Acamprosate 1998 mg/day in divided doses along with thiamine 100 mg/day. He started complaining of excessive salivation, excessive sedation, coarse tremors in hands and bradykinesia during the beginning of the second week of acamprosate therapy.

However, no scale was administered to measure the extra pyramidal syndrome (EPS). The dose of acamprosate was reduced to 1332 mg/day and continued for the next one week. There was some improvement but it was still troublesome for the patient. Finally, the drug was stopped completely and the symptoms disappeared over the next one week. There was no history of any underlying neurological illness and no sign of any neurological deficit was found. He was put on Naltrexone 50 mg/day later on for relapse prevention and he is maintaining well on this.

Although there is no clear cut evidence to suggest the emergence of these Parkinson's-like side effects and the antiglutaminergic mechanism of acamprosate, the following can be possible pathophysiological explanations:

1. The ventral midbrain dopamine (DA) neurons, which give rise to the dopaminergic pathway, may themselves be glutaminergic[6] and thus, the antiglutaminergic action of acamprosate may also block the dopaminergic pathway in the ventral midbrain.
2. The acamprosate may reduce the postalcohol cessation-induced rise in DA function, causing a hypodopaminergic state, especially in chronic alcohol-dependent individuals during the initial weeks of therapy.

This is the only one case report so one cannot make any final conclusion. But regardless of the mechanism, more research is warranted about the safety and tolerability of acamprosate in alcohol-dependent individuals and the interaction between the dopamine and glutamate pathways.
